# Efficacy and Safety of Efalizumab in Patients with Moderate-to-Severe Plaque Psoriasis Resistant to Previous Anti-Psoriatic Treatment: Results of a Multicentre, Open-label, Phase IIIb/IV Trial

**DOI:** 10.1111/j.1753-5174.2009.00026.x

**Published:** 2010-03

**Authors:** Torello Lotti, Sergio Chimenti, Andreas Katsambas, Jean-Paul Ortonne, Louis Dubertret, Daiana Licu, Jan Simon

**Affiliations:** * Department of Dermatology, University of FlorenceFlorence, Italy; † Department of Dermatology, University of Rome “Tor Vergata”Rome, Italy; ‡ Department of Dermatology, Andreas Sygros Hospital, University of AthensAthens, Greece; § CHU Hôpital l'Archet IINice, France; ¶ Hôpital St LouisParis, France; ** Merck Serono S.A.Geneva, Switzerland; †† Department of Dermatology, Venerology and Allergy, University Hospital of LeipzigLeipzig, Germany

**Keywords:** Psoriasis, Efalizumab, Treatment efficacy, Safety

## Abstract

**Objectives:**

To evaluate the efficacy and safety of efalizumab in continuous or interrupted therapy of adults with moderate-to-severe plaque psoriasis who had failed to respond to or were intolerant of other systemic therapies, including methotrexate, ciclosporin and psoralen plus UVA phototherapy, or for whom such therapies were contraindicated.

**Methods:**

Patients received a conditioning dose of efalizumab 0.7 mg/kg followed by once-weekly open-label efalizumab 1.0 mg/kg for 11 weeks. Responders (Physician Global Assessment [PGA] score of “good” or better at Week 12) could continue efalizumab for a further 8 weeks (continuous-treatment period). Nonresponders transitioned to alternative anti-psoriasis medication or stopped treatment. Responders who discontinued efalizumab could restart treatment if symptoms worsened. PGA response was evaluated at Weeks 12 (primary endpoint) and 20, as were the proportions of patients achieving an improvement from baseline of ≥50%, ≥75% and ≥90% in Psoriasis Area and Severity Index (PASI) (PASI 50, PASI 75 and PASI 90, respectively).

**Results:**

A total of 1,255 patients were included in the intention-to-treat population. At Week 12, 68.0% of patients had a PGA rating of “good” or better. Of 688 patients who entered the continuous-treatment period, 79.5% had a PGA rating of “good” or better at Week 20. At Week 12, median improvement in PASI score was 68.4%. PASI 50/75/90 was achieved by 65.5%/35.9%/13.0% of patients at Week 12, and by 78.2%/52.9%/24.3% of responders at Week 20. Of the 127 responders at Week 12 who discontinued efalizumab, 11% experienced rebound and 56.7% relapsed within 8 weeks after stopping therapy. Efalizumab was well tolerated during the study.

**Conclusions:**

Efalizumab provided effective control of psoriasis in the majority of patients during the initial treatment period. The high response rates were maintained in initial responders when treatment was continued beyond 12 weeks.

## Introduction

Psoriasis is a chronic inflammatory systemic disease [1,2], affecting between 1–3% of the population in Europe and the USA [3]. Plaque psoriasis is the most common form of the disease, accounting for more than 90% of cases [1].

Efalizumab is a recombinant humanized monoclonal immunoglobulin G1 antibody that binds to the CD11a subunit of leucocyte function-associated antigen type 1 (LFA-1). It targets multiple stages in the immunopathogenesis of psoriasis: initial T-cell activation, migration of T-cells into dermal and epidermal tissues, and T-cell reactivation [4,5]. Numerous Phase III clinical trials have demonstrated the efficacy, safety and health-related quality of life benefits of efalizumab in patients with moderate-to-severe chronic plaque psoriasis [5–11].

The current study evaluated the efficacy and safety of efalizumab in the restricted, difficult-to-treat, European-label population (adult patients with moderate-to-severe chronic plaque psoriasis who have failed to respond to or are intolerant to other systemic therapies), and the management of psoriasis rebound and exacerbation during or after efalizumab treatment. It was conducted according to the European Summary of Product Characteristics for efalizumab, which was current during the time the trial was carried out.

## Materials and Methods

### Patients

All patients were aged ≥18 years with a diagnosis of moderate-to-severe plaque psoriasis and had failed to respond, had a contraindication to, or were intolerant of other systemic therapies, including ciclosporin, methotrexate and PUVA.

Patients were required to have a white blood cell count of 4–14 × 10^9^/L and a platelet count of ≥100 × 10^9^/L. Systemic anti-psoriasis treatments were discontinued before starting study treatment with no washout period. Investigational or biological treatments for psoriasis (other than efalizumab) were also stopped at least 3 months before study treatment. Patients were not to receive any primary vaccinations within the 14 days before trial entry. Women of childbearing potential were required to use adequate contraception both during the study and for 3 months afterwards. Patients were excluded if they met any of the following criteria: the sole or predominant form of their psoriasis was guttate, erythrodermic or pustular; they had a history of severe allergic reactions to humanized monoclonal antibodies; they had withdrawn from previous efalizumab treatment as a result of lack of efficacy or an adverse event; they were pregnant or breastfeeding; they had a history of opportunistic infections or ongoing uncontrolled infections; they were seropositive for HIV, hepatitis B or hepatitis C; they had been hospitalized for cardiac disease, stroke or pulmonary disease within the last year; they had a malignancy within the past 5 years (other than fully resolved basal cell or squamous cell skin cancer). Patients with active tuberculosis (TB), a positive chest X-ray or those who had received treatment for TB within 1 year before entry were also excluded; a chest X-ray within 3 months of study treatment was required for patients considered to be at high risk for TB. Written informed consent was obtained from all patients enrolled in the trial.

### Trial Design

This was a Phase IIIb/IV, multicentre, open-label trial (study acronym CONTROL II [IMP25300]; ClinicalTrials.gov identifier NCT00249808). The trial was performed in accordance with the Declaration of Helsinki Guidelines for Good Clinical Practice, with approval by the independent ethics committee/institutional review board for each centre.

**Figure 1:**
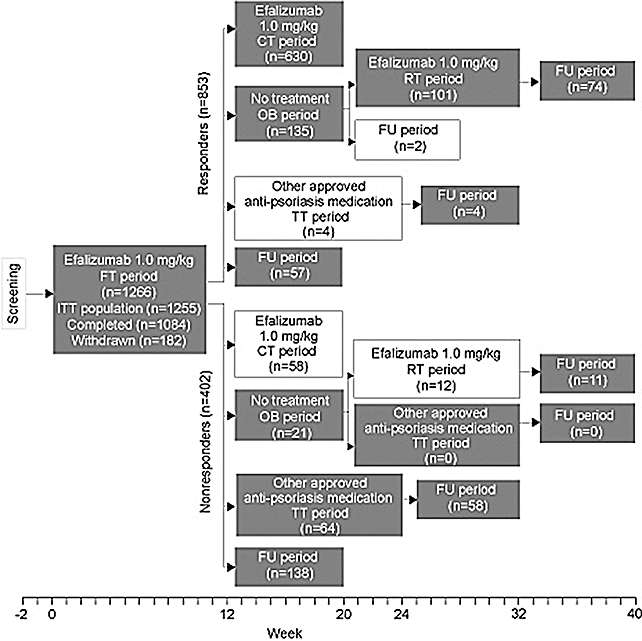
Trial design and patient disposition. White boxes indicate patients who entered inappropriate treatment or assessment periods based on their response status. CT = continuous-treatment; FT = first-treatment; FU = follow-up; ITT = intention to treat; OB = observation; RT = re-treatment; TT = transition-treatment.

The trial design is summarized in Figure 1. After a single subcutaneous (s.c.) conditioning dose of efalizumab 0.7 mg/kg, eligible patients received open-label s.c. efalizumab at a dose of 1.0 mg/kg once a week for a further 11 weeks (first-treatment period). Patients were classified at Week 12 according to the dynamic Physician Global Assessment (PGA) rating as responders (“good”, “excellent” or “cleared”) or nonresponders (“fair”, “slight”, “unchanged” or “worse”) (Table 1).

**Table 1:** Assessment criteria used to evaluate patient efficacy in this study

Assessment	Description	
Physician Global Assessment (PGA) [12,13]Global change vs. baseline in the clinical signs and symptoms of all psoriatic lesions in response to treatment (body diagrams are completed at baseline for comparison)	Cleared	100% improvement of all clinical signs and symptoms compared with baseline, except for residual manifestations (e.g., mild erythema)
	Excellent	75–99% improvement, except for residual manifestations (e.g., mild erythema)
	Good	50–74% improvement
	Fair	25–49% improvement
	Slight	1–24% improvement
	Unchanged	Clinical signs and symptoms unchanged from baseline
	Worse	Clinical signs and symptoms deteriorated from baseline
Psoriasis Area and Severity Index (PASI) [14]Extent of cutaneous psoriasis for four anatomical regions. Individual scores for each are summed, with a higher score representing more severe and extensive psoriasis	Head Trunk Upper limbs Lower limbs	Severity score: 0 (none) to 4 (very severe) for each region, for each of the following three symptoms: erythema, induration/thickness and scaling and proportion of region affected: 0 (none) to 6 (90–100% involvement)
	Total score:	0–72
	Percent improvement:	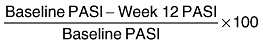

Responders could continue to receive weekly open-label s.c. efalizumab at 1.0 mg/kg for up to 8 weeks (continuous-treatment period). Nonresponders switched to treatment with an alternative approved anti-psoriasis medications, chosen by their investigator, for up to 12 weeks (transition-treatment period). Patients could choose to discontinue anti-psoriasis medication on completing the first-treatment period regardless of their response at Week 12. These patients entered the observation period and were monitored without treatment for up to 8 weeks or until signs of worsening psoriasis were observed indicating a possible relapse or rebound (in responders) or disease exacerbation (in nonresponders).

Patients who responded during the first-treatment period but experienced worsening psoriasis during the observation period could then receive further treatment with weekly open-label s.c. efalizumab at 1.0 mg/kg for 12 weeks (re-treatment period).

### Trial Assessments

PGA [12,13] and Psoriasis Area and Severity Index (PASI) [14] (Table 1) were assessed at each visit (Weeks 2, 4, 8 and 12) during the first-treatment, re-treatment and transition-treatment periods, as well as at Week 20 (continuous-treatment period) or on withdrawal from the trial. Rebound in patients who were identified as responders at Week 12 was defined as either worsening psoriasis symptoms (≥125% increase in PASI score from baseline) or the occurrence of new pustular, erythrodermic or more inflammatory psoriasis within 2 months of stopping therapy. Disease relapse was defined as a 50% reduction in the PASI improvement achieved after the initial 12 weeks of treatment with efalizumab.

Disease exacerbation was defined in nonresponders (i.e., patients with PGA ratings of “fair”, “slight”, “unchanged” or “worse” either during or after treatment) as worsening of disease to a more inflammatory state than at baseline, and that occurred within pre-existing plaques, at previously uninvolved sites, or as new morphologies of the disease.

Adverse events were assessed at every study visit throughout the trial (Weeks 0, 2, 4, 8, 12 and 20). Adverse events were coded using MedDRA (version 8.1). Reported adverse events were classified as pre-first-treatment period, first-treatment-emergent or post-first-treatment period events based on their onset dates and the dates of first and last “first-treatment period” treatment administration. The severity of adverse events was assessed as mild (the patient was aware of the event or symptom but it was easily tolerated), moderate (the patient experienced sufficient discomfort to interfere with, or reduce, his or her usual level of activity) or severe (the patient experienced a significant impairment of functioning and was unable to carry out his or her usual activities). A serious adverse event was defined as an event resulting in death or that was life-threatening, required hospitalization or prolongation of existing hospitalization, resulted in persistent or significant disability or incapacity, was a congenital anomaly or birth defect, or was a medically important condition (the event did not have to be immediately life-threatening or result in death or hospitalization, but was clearly of major clinical significance).

### Statistical Considerations

The primary efficacy endpoint was PGA response at Week 12, with outcomes also assessed at Week 20. Patients with missing PGA assessments were classified as nonresponders (worst-case imputation). The proportions of patients with a decrease of ≥50%, ≥75% and ≥90% in PASI score from baseline (PASI 50, PASI 75 and PASI 90 response, respectively) were calculated at Weeks 12 and 20.

Analyses were conducted according to treatment period for the intention-to-treat (ITT) population, which included all patients who received at least one dose of trial treatment and who underwent at least one post-treatment efficacy assessment. Analyses for the safety population, which included all patients who received at least one dose of trial treatment, included analyses of adverse events, safety laboratory outcomes and vital signs. Patients on continuous treatment who received efalizumab in accordance with the protocol (i.e., those who had achieved a PGA response of “good” or better at first-treatment Week 12) were evaluated as a subset of the whole continuous-treatment population. Analyses for this trial were primarily descriptive in nature and no formal hypothesis testing was performed. Exact 95% confidence intervals (CIs) for proportions were obtained using the PROC FREQ procedure from SAS. The PROC LIFETEST procedure from SAS was used to generate Kaplan–Meier estimates of time to rebound and disease relapse.

## Results

### Patients

The trial was conducted at 170 centres across 18 European countries. Between 13 December 2004 and 12 April 2006, 1,266 patients were enrolled in the trial.

Patient disposition is shown in Figure 1. The ITT population comprised 1,255 patients, as 11 patients were excluded because no post-baseline efficacy assessment data were recorded for them during the first-treatment period. A total of 688 patients continued into the efalizumab continuous-treatment period after Week 12, of whom 630 met the eligibility criterion for a PGA “good” or better treatment response. The remaining 58 patients were nonresponders who continued to receive efalizumab during the continuous-treatment period despite it being a deviation from the trial protocol. Data collected from these patients were also analyzed as part of this study. Sixty-eight patients transitioned to other anti-psoriasis treatment after Week 12 (4 responders and 64 nonresponders) and 156 patients (135 responders and 21 nonresponders) did not wish to continue treatment and went into the observation period, while 195 patients (57 responders and 138 nonresponders) entered a follow-up period. After Week 12, 148 patients (27 responders and 121 nonresponders) had no follow-up. The baseline demographics for the ITT population are summarized in Table 2. Almost half of the patients had a baseline PASI score > 20. Prior medications for psoriasis included immunosuppressive agents (ciclosporin, infliximab, mycophenolate mofetil, efalizumab, etanercept, methotrexate), retinoids (acitretin, etretinate), phototherapy (UVB, systemic and topical PUVA) and topical anti-psoriatic medications.

**Table 2:** Baseline patient demographics and disease characteristics

Characteristic	ITT population (N = 1,255)
Demography	
Median age, years (range)	46.0 (18–81)
Sex, N (%)	
Male	860 (68.5)
Race, N (%)	
White	1,233 (98.2)
Black	6 (0.5)
Asian	10 (0.8)
Other	6 (0.5)
Median weight, kg (range)	80.0 (45.0–160.7)
Median body mass index, kg/m^2^ (range)*	26.7 (16.4–64.4)
Disease characteristics	
Median duration of psoriasis, years (range)†	18.7 (0.7–66.1)
Patients with prior psoriasis therapy, N (%)	1,243 (99.0)
Patients with prior systemic therapy, N (%)	1,218 (97.1)
Patients receiving medication prior to informed consent, N (%)§	558 (44.1)
Ciclosporin	252 (19.9)
Acitretin	143 (11.3)
Methotrexate	106 (8.4)
Calcipotriol	47 (3.7)
Dovobet/01643201	21 (1.7)
Daivobet/01643401	18 (1.4)
Psoralens for systemic use	51 (4.0)
Clobetasol propionate	17 (1.3)
Median PASI score (range)¶	19.6 (0.7–67.2)
PASI score ≥ 20‡, N (%)	611 (48.7)

* N = 1,233;

† N = 1,252;

‡ N = 1,266, safety population;

§ Medications received by >1% of patients;

¶ N = 1,254.

ITT = intention to treat; PASI = Psoriasis Area and Severity Index.

### Efficacy with Continuous Efalizumab Treatment

#### Physician Global Assessment

**Figure 2:**
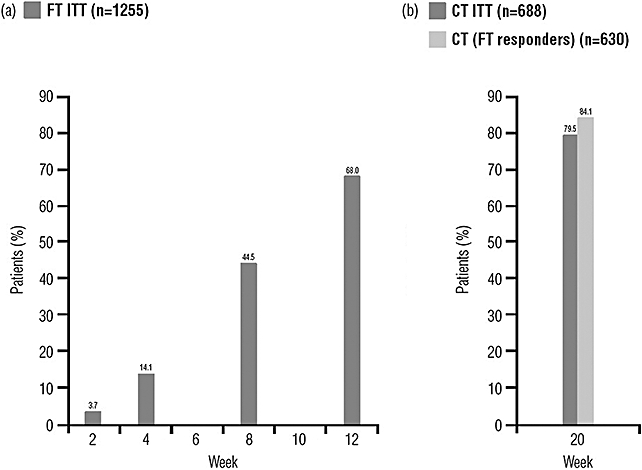
Proportions of patients with a PGA rating of “good”, “excellent” or “cleared” by visit at (a) Week 12, after FT period (ITT population) and (b) Week 20, after CT period. Of the CT ITT population, 630 patients had responded during the FT period (FT responders), whereas the remaining 58 patients were nonresponders who had continued to receive efalizumab during the CT period. CT = continuous-treatment; FT = first-treatment; ITT = intention to treat; PGA = Physician Global Assessment.

The proportion of patients in the ITT population (N = 1,255) with a PGA rating of “good”, “excellent” or “cleared” increased throughout the first-treatment period, reaching 68.0% by Week 12 (N = 853; 95% CI: 65.3–70.5%; Figure 2a).

Among those who entered into the continuous-treatment period (N = 688; ITT population), 79.5% (N = 547; 95% CI: 76.3–82.5%) had a PGA rating of “good”, “excellent” or “cleared” at Week 20 (Figure 2b).

The ITT population for the continuous-treatment period included the 58 nonresponder patients mentioned previously who, in a deviation from the trial protocol, continued to receive efalizumab up to Week 20. Of these, 29.3% (N = 17) achieved a PGA rating of “good”, “excellent” or “cleared” by Week 20.

#### Psoriasis Area and Severity Index

**Figure 3:**
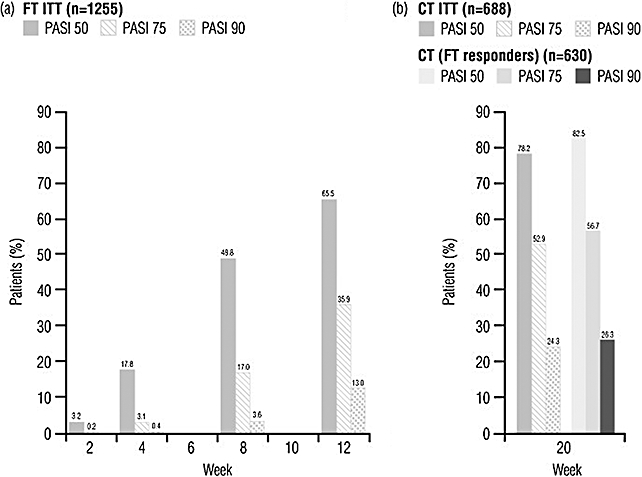
Proportions of patients with PASI 50, PASI 75 and PASI 90 responses during the FT and CT periods (a) by visit for the ITT population during the FT period and (b) at Week 20 after the CT period. Of the CT ITT population, 630 patients had responded during the FT period (FT responders), whereas the remaining 58 patients were nonresponders who had continued to receive efalizumab during the CT period. CT = continuous-treatment; FT = first-treatment; ITT = intention to treat; PASI = Psoriasis Area and Severity Index.

By Week 12, the median improvement from baseline in PASI score in the ITT population was 68.4% (median PASI score at baseline of 19.55 and 6.00 at Week 12). The proportion of patients in the ITT population with a PASI 50/PASI 75/PASI 90 response increased throughout the first-treatment period to 65.5%/35.9%/13.0% at Week 12 (Figure 3a).

At Week 20, the median PASI score was 3.90 for patients in the continuous-treatment period and the proportion of patients in the ITT population with a PASI 50/PASI 75/PASI 90 response increased further through the continuous-treatment period to 78.2%/52.9%/24.3% of responders at Week 20 (Figure 3b).

### Efficacy of Interrupted Efalizumab Treatment or Transition to Alternative Anti-Psoriasis Medication

**Figure 4:**
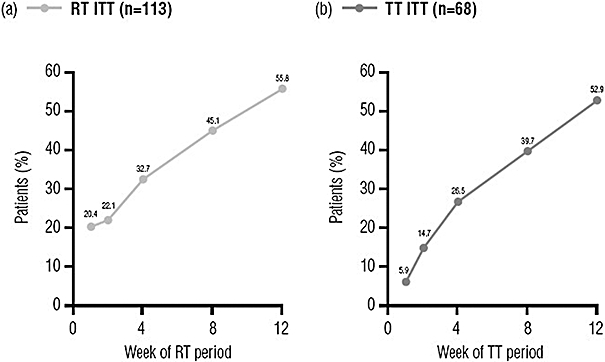
Proportions of patients with a PGA rating of “good”, “excellent” or “cleared” by visit after (a) re-treatment with efalizumab (RT period*; ITT population, N = 113) or (b) transition to an alternative anti-psoriasis agent (TT period^†^; ITT population, N = 68). *Patients had completed the FT period and responded but opted to enter the OB period rather than continue treatment with efalizumab; efalizumab treatment was restarted when their psoriasis began to worsen during the OB period. ^†^Patients had completed the FT period but had not responded and received treatment with another approved anti-psoriasis medication. FT = first-treatment; ITT = intention to treat; OB = observation; PGA = Physician Global Assessment; RT = re-treatment; TT = transition-treatment.

Of the 135 responders who entered the observation period and therefore had their efalizumab therapy suspended for 8 weeks, 102 subsequently entered the re-treatment period due to worsening signs of psoriasis during the observation period. The proportion with a PGA rating of “good”, “excellent” or “cleared” increased from 20.4% at Week 1 of the re-treatment period to 55.8% at Week 12 (Figure 4a).

The proportion of patients who transitioned to other anti-psoriasis treatment at Week 12 (N = 68) and who were identified as having a PGA rating of “good”, “excellent” or “cleared” also increased across the transition-treatment period, from 5.9% at Week 1 to 52.9% by Week 12 (Figure 4b). Response rates at Week 12 of the transition-treatment period were highest among patients who transitioned to ciclosporin (N = 37; 64.9% with a rating of “good”, “excellent” or “cleared”). It should be noted, however, that most patients received more than one treatment during this period.

#### Rebound and Relapse

In total, 135 of 853 first-treatment responders entered the observation period. Out of the 127 evaluable patients, 56.7% (N = 72) relapsed, with a median time to relapse of 56 days, and 11% (N = 14) of the evaluable patients experienced rebound during the observation period.

#### Disease Exacerbation

Of the 402 first-treatment nonresponders, 37 (9.2%) experienced disease exacerbation during the first-treatment period. A total of 21 first-treatment nonresponders entered the observation period and 64 entered the transition-treatment period—treatment with an alternative approved anti-psoriasis medication. Of these, 4.8% (N = 1) and 3.1% (N = 2) experienced new or additional exacerbation of their psoriasis during the observation and transition-treatment periods, respectively. Overall, 12.9% (N = 52) of nonresponders experienced disease exacerbation at some point during the trial.

### Safety

#### Exposure

The median duration of exposure to efalizumab in the first-treatment period was 78 days (range: 1–134 days); two patients received efalizumab for >100 days. The median cumulative dose was 11.7 mg/kg (range: 1–18 mg/kg). The median duration of exposure to efalizumab during the continuous-treatment and re-treatment periods was 57 days (range 1–266 days) and 78 days (range 1–373 days), respectively.

#### Treatment-Emergent Adverse Events

Treatment-emergent adverse events (TEAEs) are summarized in Table 3. The most common TEAEs in the first-treatment period were headache (25.5% of patients), pyrexia (9.6% of patients) and influenza-like illness (8.5% of patients), with most being of mild or moderate severity (2,197/2,371 events, 92.7%). Severe TEAEs occurred in 125 patients (9.9%), the most common being headache (31 events), psoriasis (18 events) and pyrexia (10 events). The incidence of TEAEs of all severities was lower during the continuous-treatment period than during the first-treatment period (Table 3); the most common TEAEs were psoriasis (1.7% of patients), arthralgia (1.0%), nasopharyngitis (0.9%) and pyrexia (0.9%).

**Table 3:** Treatment-emergent adverse-event (TEAE) summary of the safety population during efalizumab treatment

	Number of patients (%)		
	FT period (N = 1,266)	CT period (N = 688)	RT period (N = 113)
Any TEAE	785 (62.0)	122 (17.7)	46 (40.7)
Serious TEAE	60 (4.7)	10 (1.5)	3 (2.7)
TEAE leading to withdrawal*	89 (7.0)	4 (0.6)	4 (3.5)
Nervous system disorders	355 (28.0)	9 (1.3)	3 (2.7)
General disorders and administration-site conditions	318 (25.1)	10 (1.5)	5 (4.4)
Infections and infestations	212 (16.7)	35 (5.1)	19 (16.8)
Musculoskeletal and connective tissue disorders	176 (13.9)	21 (3.1)	14 (12.4)
Skin and subcutaneous tissue disorders	170 (13.4)	32 (4.7)	9 (8.0)
Gastrointestinal disorders	138 (10.9)	7 (1.0)	4 (3.5)
Malignancy	4 (0.3)†	0 (0)	0 (0)‡
Death	1 (0.1)§	0 (0)	0 (0)

* TEAEs leading to withdrawal from the study were not necessarily from the period in which they emerged.

† Three were considered unrelated or unlikely to be related and one possibly related to study treatment.

‡ One malignancy, cutaneous squamous cell carcinoma reported during the follow-up period after RT 6 weeks after the last efalizumab dose, was considered possibly related to study treatment.

§ Cardiopulmonary failure (deemed unlikely to be related to trial treatment).

CT = continuous-treatment; FT = first-treatment; RT = re-treatment.

Twenty-two of the 68 patients (32.4%) who received other approved psoriasis therapies (including but not limited to retinoids, fumarates, steroids, methotrexate, ciclosporin, PUVA and UVB) during the transition-treatment period reported TEAEs. Among those events reported by ≥5% of patients, musculoskeletal disorders (11.8%) and skin disorders (8.8%) were the most common, followed by fatigue, feeling cold, influenza-like illness and pyrexia (each 5.9%).

During the re-treatment period, a total of 77 TEAEs were reported in 40.7% of patients. The most common TEAEs reported during the re-treatment period were nasopharyngitis (8.8%), arthralgia (3.5%) and back pain (3.5%).

#### Serious TEAEs

Fewer than 5% of patients experienced serious TEAEs during the first-treatment period, with lower incidences reported during the continuous-treatment and re-treatment periods (Table 3). The most common serious events were psoriasis (1.1%), psoriatic arthropathy (0.5%) and pyrexia (0.3%). Serious TEAEs during the first-treatment period are summarized in Table 4.

**Table 4:** Serious treatment-emergent adverse events (TEAEs) in the safety population during the first-treatment period with efalizumab

	Number of patients (%) (N = 1,266)
Any serious TEAEs	60 (4.7)
Skin and subcutaneous tissue disorders	21 (1.7)
Musculoskeletal and connective tissue disorders	10 (0.8)
Infections and infestations	10 (0.8)
General disorders and administration-site conditions	7 (0.6)
Cardiac disorders	6 (0.5)
Gastrointestinal disorders	5 (0.4)
Nervous system disorders	5 (0.4)
Immune system disorders	2 (0.2)
Injury, poisoning and procedural complications	2 (0.2)
Malignancy	2 (0.2)
Ear and labyrinth disorders	1 (0.1)
Eye disorders	1 (0.1)
Hepatobiliary disorders	1 (0.1)
Psychiatric disorders	1 (0.1)
Respiratory, thoracic and mediastinal disorders	1 (0.1)

One patient, a 71-year-old man, died during the trial. This patient had received a single dose of efalizumab before being withdrawn because of an upper respiratory infection and fever. A month later he experienced fatal cardiopulmonary failure. This event was considered by the investigator as unlikely to be related to trial medication.

#### Other Significant TEAEs

During the first-treatment period 89 subjects (7.0%) had 134 adverse events that led to treatment discontinuation. Of these events, the most frequent were due to skin and s.c. tissue disorders, general disorders and administration-site conditions, and musculoskeletal and connective tissue disorders (Table 5).

**Table 5:** Treatment-emergent adverse events (TEAEs) leading to withdrawal in the safety population during the first-treatment period with efalizumab

	Number of patients (%) (N = 1,266)
Any TEAE leading to withdrawal	89 (7.0)
Skin and subcutaneous tissue disorders	30 (2.4)
General disorders and administration-site conditions	22 (1.7)
Musculoskeletal and connective tissue disorders	17 (1.3)
Infections and infestations	12 (0.9)
Gastrointestinal disorders	9 (0.7)
Nervous system disorders	8 (0.6)
Investigations	4 (0.3)
Cardiac disorders	4 (0.3)
Blood and lymphatic system disorders	3 (0.2)
Immune system disorders	3 (0.2)
Vascular disorders	3 (0.2)
Psychiatric disorders	2 (0.2)
Ear and labyrinth disorders	1 (0.1)
Eye disorders	1 (0.1)
Metabolism and nutrition disorders	1 (0.1)
Malignancy	1 (0.1)

Four malignancies were reported during the first-treatment period: two men (aged 57 and 44 years) with basal cell carcinoma, a 49-year-old woman and a 43-year-old man with squamous cell carcinoma. Only the last case was considered to possibly be related to trial treatment. The patient in question had a history of squamous and basal cell carcinoma. An additional cutaneous squamous cell carcinoma was reported during follow-up. This occurred 6 weeks after the last efalizumab dose given during the re-treatment period and was considered to possibly be related to study treatment.

The most frequently reported infection-related TEAEs during the first-treatment period were nasopharyngitis (3.2%), influenza (2.2%) and herpes simplex (1.9%). Infections and infestations were the most common TEAEs in the continuous-treatment and re-treatment periods (Table 3) and accounted for 10 (0.8%) of the serious TEAEs reported during the first-treatment period.

With the exception of two cases of severe influenza in two patients (0.1%) and two cases of severe bronchitis in two patients (0.1%), no other severe infections occurred in more than one patient. There were no reports of TB, opportunistic infection, demyelinating disease or serious thrombocytopenia in any patients during this trial.

## Discussion

This prospective, post-approval clinical trial assessed the efficacy and safety of efalizumab therapy in patients across Europe with moderate-to-severe chronic plaque psoriasis who had failed to respond to, had contraindications for, or were intolerant of other systemic therapies. The main strengths of this study were the large patient cohort and the unique study design, which enabled a more accurate reflection of routine clinical practice by allowing patients receiving other anti-psoriasis drugs, those not receiving any treatment, and those restarting treatment to continue follow-up.

Results obtained from the initial continuous-treatment section of the trial show that over two-thirds of patients had responded by Week 12 of efalizumab treatment and this high response rate was maintained in responders who continued treatment for up to 20 weeks. These findings are consistent with data obtained from previous pivotal clinical trials, the open-label LATAM trial and longer-term studies [6,8–11,15].

Following this initial 12-week treatment period, the findings from this study indicate that efalizumab treatment can be discontinued, if alternative therapy is initiated in its place. Of the responders who stopped efalizumab treatment at the end of the initial 12-week period and did not transition to an alterative therapy, 11% experienced rebound. Responding patients who had discontinued and then restarted efalizumab treatment were found to achieve a similar (but slightly reduced) efficacy and tolerability as attained during the initial treatment period.

This trial also explored treatment options for patients who do not initially respond to efalizumab therapy. More than half of the nonresponders who entered the transition-treatment period subsequently achieved a response after changing to another anti-psoriatic therapy.

The major limitation of this study was the open-label, noncomparative trial design. Indeed, the range of anti-psoriatic therapies permitted across the transition-treatment period, while an accurate reflection of real-life clinical experience, complicates the analysis of treatment effectiveness during this time. It is also difficult to interpret the data obtained during the observation period, since few nonresponders entered this section of the study.

The incidence of psoriasis exacerbation in nonresponders was carefully monitored, but found to be lower after the initial 12-week treatment period, indicating that the discontinuation of efalizumab in these patients was not associated with psoriasis-related adverse events. Due to the immunomodulatory mechanism of action of efalizumab, the incidences of infections and malignancies in treated patients were also followed closely. During this trial the safety of efalizumab was found to be acceptable and consistent with previous results. No new safety issues were identified. The incidence of TEAEs was lower in the continuous-treatment period than in the initial-treatment period, with influenza-like symptoms being the most frequent TEAEs in the first-treatment period, as observed in previous studies [5–10]. Unlike trials examining tumour necrosis factor blocking agents, no increase in the risk of TB with treatment with efalizumab was observed [16]. Four malignancies were reported during the first-treatment period but the number of cases in this study was not sufficient to draw any firm conclusions regarding the risk of malignancy with this drug.

No TEAEs related to opportunistic infections were observed during this trial. However, opportunistic infections have been reported in the post-marketing surveillance in patients with psoriasis receiving efalizumab. In particular, cases of JC virus infection resulting in progressive multifocal leucoencephalopathy have been reported in patients receiving efalizumab continuously for more than 3 years. After evaluating all available safety data, the European Medicines Agency concluded that the benefits of efalizumab treatment no longer outweighed the risks associated with the drug and recommended suspension of marketing authorization on 19 February 2009.

This study addressed important questions relating to the appropriate management of both responding and nonresponding psoriasis patients in real-life clinical situations, beyond the initial 12-week treatment period. Efalizumab was found to provide effective control of psoriasis in two-thirds of patients within 3 months of treatment. Of 127 evaluable patients who entered the observation period, more than half relapsed and 11% (N = 14) experienced rebound during the observation period. However, the greatest therapeutic benefit was obtained by initially responding patients who were given continuous efalizumab-treatment for the full 5-month trial period.
